# Integrating dorsal root ganglion stimulation with transforaminal lumbar interbody fusion: proof of concept study

**DOI:** 10.1016/j.xnsj.2026.100906

**Published:** 2026-06-01

**Authors:** Michael C Park, Rohan R Lall, Deepak Reddy, Jonathan N Sembrano, Steven M Falowski, Nameer R Haider

**Affiliations:** a Department of Neurosurgery, University of Minnesota, 420 Delaware St SE, MMC 96, Minneapolis, MN 55455, United States; b Department of Neurosurgery, MHealth Fairview, 6545 France Ave S, Edina, MN 55435, United States; c South Bend Orthopaedics, 53880 Carmichael Drive, South Bend, IN 46635, United States; d Department of Orthopedic Surgery, University of Minnesota, 2512 South 7th St, Suite R200, Minneapolis, MN 55454, United States; e Advanced Surgery Center of Lancaster, 160 N. Pointe Boulevard #200, Lancaster, PA 17601, United States; f Omni Pain and Precision Medicine, 110 Business Park Drive, Utica, NY 13502, United States

**Keywords:** Chronic lower back pain, Lumbosacral radiculopathy, Failed back surgery syndrome, DRG stimulation, Lumbosacral fusion, TLIF, Direct Visual Placement, Back pain VAS score, Leg pain VAS score, Opioid usage

## Abstract

**Background:**

Transforaminal lumbar interbody fusion (TLIF) is a modality for treatment of chronic lower back pain (CLBP) and/or lumbosacral radiculopathy (LR). Up to 28% of patients with indications for surgical treatment of CLBP/LR continue to have CLBP/LR after surgery despite a technically successful spinal surgery, likely due to development or persistence of neuropathic pain. Dorsal root ganglion (DRG) stimulation is often used for the treatment of chronic neuropathic pain. Thus, we present a novel technique of integrating DRG stimulation using Direct Visual Placement with open lumbar or lumbosacral decompression and instrumented fusion in a single surgical procedure.

**Methods:**

In an uncontrolled case series for a proof of concept study, we combined DRG stimulation with TLIF in a single surgical procedure to evaluate feasibility and safety. Fifteen patients with CLBP/LR and indication for TLIF received DRG stimulator placement concomitantly during TLIF. Safety of the combined approach, namely adverse event (AE), was evaluated. Also, change in back pain and leg pain visual analog scale (VAS) scores, as well as opioid usage, from baseline to 12 weeks, 6 months, and 12 months postoperatively, was tracked.

**Results:**

No AE was serious, and was related to the 2 procedures, TLIF and DRG stimulation, being combined. One instance of electrode migration without loss of therapy was recorded. Stimulation parameters were 20 Hz frequency, 250 microsecond pulse width, and less than 1 mA amplitude. Back pain VAS scores improved 67%, 72%, and 71% from baseline at 12 weeks, 6 months, and 12 months, respectively. Leg pain VAS scores improved 67%, 69%, and 69% from baseline at 12 weeks, 6 months, and 12 months, respectively. Responder (VAS score reduction of at least 50%) analysis of back pain and leg pain VAS scores showed that 53.3% and 60.0% of subjects responded to stimulation at 10-20 days for back pain and leg pain, respectively. Responder rates increased to 66.7% and 73.3% at 12 months for back pain and leg pain, respectively. Responder rates for “either” or “both” were 80.0% and 66.0% at 12 months, respectively. Opioid usage was down to 20% from 12 weeks to 12 months.

**Conclusions:**

This series demonstrates the feasibility of integrating DRG stimulator placement with open lumbar or lumbosacral decompression and instrumented fusion. The Direct Visual Placement of DRG stimulator demonstrated no TLIF and DRG stimulation combination related AEs, showed feasibility, reduced pain and improved quality of life of the subjects, thus presenting a new treatment approach for CLBP/LR in a single procedure.

## Introduction

Chronic lower back pain (CLBP) and lumbosacral radiculopathy (LR) are caused by common problems such as degenerative disc disease, degenerative facet disease, fractures, spinal stenosis, and spondylolisthesis, and often lead patients to undergo spinal decompression and fusion procedures to address the anatomical problems for pain relief. There are approximately 500,000 low-back fusion procedures performed annually in the US. However, spinal fusion procedures do not always relieve pain as intended despite a successful surgical decompression and fusion. In patients with CLBP and LR, there are often both nociceptive and neuropathic pain components which can complicate patient selection and lead to suboptimal functional and pain outcomes. Studies identified between 20% and 47% of patients with low back pain experience neuropathic symptoms prior to surgery [1,2]. Up to 28% of patients with indications for surgical treatment of LR may additionally develop chronic back or leg pain after surgery, likely due to development or persistence of neuropathic pain [3]. Furthermore, patients with preoperative neuropathic pain have been found to have smaller improvements in pain after lumbar fusion [4].

Residual pain after a successful spinal fusion is so common that it is referred to as failed back surgery syndrome (FBSS) and occurs after up to 40% of spinal surgeries [5]. FBSS costs an average of $20 billion US health care dollars per year and severely reduces quality of life for patients [6]. Furthermore, patients often receive physical therapy or are prescribed opioids to combat pain associated with FBSS, which has contributed to the current opioid epidemic [7]. In some instances, patients undergo additional low-back fusion procedures.

Patients with FBSS who fail conservative treatments but are not candidates for further surgical intervention are often treated with neuromodulation. Treatment with spinal cord stimulation (SCS) prior to high dose analgesics or additional surgery was found to have better outcomes [8]. Recently, new targets for stimulation have been found to be advantageous and effective for pain relief, including the dorsal root ganglion (DRG) [9,10]. While neuromodulation is an effective treatment for neuropathic pain relief, most patients with FBSS do not receive the therapy until 5.45 years after their initial spinal fusion, resulting in prolonged disability and morbidity [11]. Unfortunately, delays in receiving neuromodulation therapy are associated with worse pain outcomes [11]. Access to DRG stimulation is further limited due to postsurgical scar formation which eliminates the traditional percutaneous epidural approach to lead placement. However, alternative techniques, including open placement of DRG leads, have been reported for patients who have scar formation secondary to previous spinal procedures [12,13].

To address the problem of CLBP and LR consisting of both nociceptive and neuropathic pain, we propose a novel approach of integrating 2 established therapies in a single procedure, open lumbar or lumbosacral decompression and instrumented fusion with DRG stimulation, to treat all the components of pain. Immediately after the standard of care open back spinal fixation procedure and prior to closure, the surgeon will have direct open access to the neural foramen to place a DRG stimulator system. This differs from the on-label DRG stimulator implantation method as it is conducted under direct vision, Direct Visual Placement, and during the same surgical procedure as implantation of the spinal fixation system, instead of separately in time and via the traditional percutaneous epidural fashion. We hypothesize that this combined procedure will be safe, easy to implement and pose minimal added risk.

## Methods

### Study design and inclusion criteria

The study is a prospective single arm proof of concept study, uncontrolled case series, evaluating the safety and tolerability of a novel procedure of combining surgical spinal fusion and DRG neuromodulation (e-TLIF). The device used in the study is a commercially available DRG stimulator system that is approved for a different indication and is therefore considered investigational use in this study. The DRG stimulator system includes an implantable pulse generator (IPG) that generates mild electrical pulses and allows for connection to up to 4 leads. Each lead has 4 electrode contacts at the distal end used to deliver current to the intended neural target.

Eligible patients had to be at least 21 years old, be diagnosed with chronic pain of the lower back and/or leg, visual analog scale (VAS) score ≥5, which has been refractory to conservative therapy for at least 3 months, be indicated for a 1 or 2 level spinal fusion, open back transforaminal lumbar fusion surgery with pedical screw fixation with or without interbody cage, and be a candidate for neuromodulation. The Institutional Review Board approved the study and written informed consent was obtained from all participants.

### Surgical technique

All study participants underwent standard of care open back spinal fixation procedure using a transforaminal lumbar interbody fusion (TLIF) approach at the lumbar (L) 3 to sacral (S) 1 levels, 1 or 2 levels, with optional use of an interbody cage. The DRG stimulator system is implanted following the fusion procedure but prior to wound closure.

**Fig. 1:**
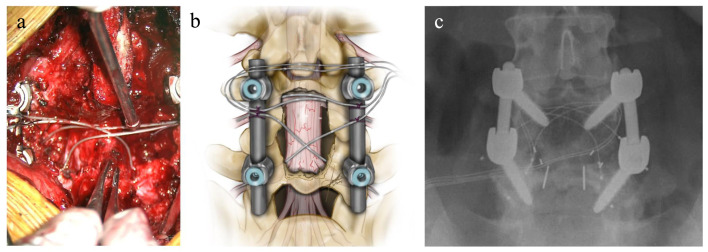
Intraoperative (A), illustrative (B), and radiographic (C) views of the implant site showing 4 dorsal root ganglion stimulator leads in the lumbar spine placed concurrently with spinal fusion hardware.

For the placement of the DRG leads, the anchoring sutures, 3-0 Vicryl sutures, are placed around and tied to each of the rods near the caudal pedicle screw (Fig. 1). Three knots are placed for each anchoring suture. If 2 leads are planned for each side, the 2 anchoring sutures are spaced out by approximately 1 cm. Using a Woodson dental instrument, the neural foramen is first palpated to confirm adequate decompression and that there is sufficient space dorsal to the nerve root. The lead is inserted into the foramen under direct vision, Direct Visual Placement, and placed at the dorsal aspect of the nerve root with the most proximal contact visible and contacting the nerve root. The lead is then oriented relatively straight and tied down with the anchoring suture on the rod contralateral to the neural foramen. Once secured, the inner stylet is removed from the lead. Each of the leads is placed using the same technique and intraoperative fluoroscopy used to confirm lead placement.

Then, the IPG was placed and connected to the electrodes. A horizontal incision is made over the right or left buttock area for the IPG implant which is deepened until the subcutaneous fatty layer is reached and a pocket created approximately 1 cm below the skin in the inferior direction to the incision. The leads are then tunneled from the lumbar incision to the pocket. The passage is from the subfascial space in the lumbar area to the subcutaneous space in the buttock. Care is taken not to kink or bend the lead from the anchoring points to the IPG site. Once tunneled, the distal leads are gently pulled until there is no more excess lead length within the lumbar wound. The connector end of each lead is then inserted into the IPG and secured. The IPG is placed within the pocket, with care being taken to place the excess wires behind and around the periphery of the device. The IPG is then anchored to the pocket using two 2-0 Ethibond sutures.

Fluoroscopy images are taken to confirm that the lead placement is satisfactory and that each manipulation did not result in any movement of the electrodes within the foramina. The buttock incision is reapproximated in the typical fashion in layers. With the DRG stimulator system implanted, lumbar fusion surgery can resume to posterolateral fusion and final wound closure.

### Clinical assessment and follow-up

The primary safety endpoint was to characterize device-, surgical procedure-, stimulation therapy-, and/or TLIF and DRG stimulation combination-related adverse event (AE). The primary safety endpoint of the study was tracked throughout the study up to 12 months. After the surgical implantation, patients underwent clinical assessment related to the safety of the combined approach including AE related to the device, surgical procedure, stimulation therapy, and/or TLIF and DRG stimulation combination. Radiographic analysis of the device position was also performed during regularly scheduled follow-up visits up to 12 months.

Primary efficacy endpoint was defined as change in back and leg pain VAS scores from baseline to 12 weeks postoperatively. The back pain and leg pain VAS scores, were tracked throughout the study, from baseline to 12 weeks, 6 months, and 12 months postoperatively. Secondary endpoint was defined as the change in neurological status, disability, quality of life, and use of analgesics, from baseline to 12 weeks postoperatively. The neurological status, disability, quality of life, and use of analgesics were tracked throughout the study, from baseline to 12 weeks, 6 months and 12 months postoperatively.

## Results

### Study population and operative technique

Fifteen participants (10 female) with average age of 54.5 years and 7.3 years of CLBP were enrolled in the study and underwent placement of a DRG stimulator during the open lumbar or lumbosacral decompression and instrumented fusion surgery from January 1, 2022 to October 11, 2024 (Table 1). The patients underwent L4–5 (6/15, 40%), L5–S1 (4/15, 26.6%), L3–5 (3/15, 20%), or L4–S1 (2/15, 13.3%) decompression and instrumented fusion and had up to 4 total DRG electrodes placed (Table 2).

**Table 1:** Patient baseline characteristics.

Characteristic	Mean ± SD (n) or n/N (%)
Age (year)	54.5 ± 11.22 (15)
Female gender	10/15 (66.7%)
Race: White or Caucasian	12/15 (80%)
Neurological examination: Normal	15/15 (100.0%)
Duration of chronic low back pain (year)	7.28 ± 6.907 (15)
VAS score for back pain	7.06 ± 1.265 (15)
VAS score for leg pain	6.83 ± 1.131 (15)
ODI score (%)	52.6 ± 12.60 (15)
EQ-5D-5L Index Value	0.322 ± 0.2512 (15)

EQ-5D-5L, Five-level Euroqol Five-dimensional Questionnaire; ODI, Oswestry Disability Index, VAS, Visual Analog Scale.

**Table 2:** Summary of the surgical procedures.

Procedure Information	Mean ± SD (n) or n/N (%)
Level of fusions for TLIF—n/N (%)	
One-level	10/15 (66.7%)
Two-levels	5/15 (33.3%)
Levels of fusion—n/N (%)	
L4–L5	6/15 (40.0%)
L5–S1	4/15 (26.6%)
L3–L5	3/15 (20.0%)
L4–S1	2/15 (13.3%)
Lead location—DRG level	
L3	4/58 (6.9%)
L4	21/58 (36.2%)
L5	23/58 (39.7%)
S1	10/58 (17.2%)

DRG, dorsal root ganglion; L, lumbar; S, sacral; TLIF, transforaminal lumbar interbody fusion.

**Fig. 2:**
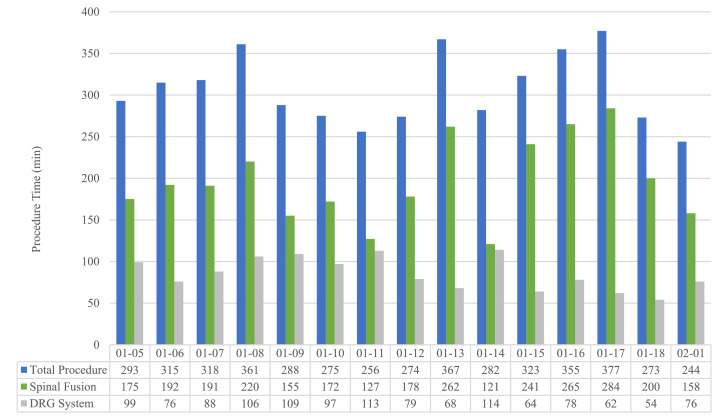
The procedure times by implanted subject. Four leads were implanted in all subjects except subject 01-06 who only had 2 leads implanted. The mean dorsal root ganglion stimulator (DRG system) implant time, even including a 2-lead case, was longer in the first 7 cases than the last 8 cases (98.3 vs. 74.4 minutes). Two-level fusion (subjects 01–13, 01–15, 01–16, 01–17 and 02–01) attributed to a longer total procedure time (333.2 vs. 293.5 minutes) and spinal fusion time (242.0 vs. 173.1 minutes) compared to 1-level fusion procedure, but not the DRG system implant time (69.6 vs. 93.5 minutes) since they were all later cases.

The incremental time added to the lumbar fusion procedure related to DRG stimulator placement was evaluated separately for the lead and IPG placement. Operative times varied based on anatomical considerations of each patient, procedural learnings, and operating room workflow considerations (Fig. 2). The average total operative time for the integrated DRG stimulator placement with open lumbar or lumbosacral decompression and instrumented fusion was 306.7 ± 42.4 minutes (range 244–377 minutes), with the average operative time related specifically to the placement of the DRG stimulator being 72.4 ± 15.9 minutes (range 46–101 minutes).

Two adaptations to the DRG stimulator placement technique were made after the first implantation surgery. First, the DRG electrodes were taken out of the introducing sheath provided with the DRG stimulator kit to allow free passage with improved manipulation of the array within the foramen. Second, we opted to place a second pair of DRG electrode arrays, leaving 4 arrays covering bilateral DRGs at the level of the fusion and the level below the fusion. There were no intraoperative complications including those related to the DRG stimulator device.

### Stimulation settings

All subjects had neurostimulator therapy turned on by the technical representative from the sponsor within 24 hours postoperatively. Per the protocol, programming for all subjects included a fixed pulse width of 250 μsec and a frequency of 20 Hz. The amplitude was programmed at approximately 20% below paresthesia levels to cover the subject’s pain areas.

One subject (01–06) had 2 leads implanted bilaterally while all other subjects had 4 leads implanted bilaterally. Table 3 summarizes lead electrical parameters through 12 months. The mean amplitude at 24 hours post-implant was 0.49 mA; it was slightly increased but stabilized throughout 12 months. It was 0.7 mA and 0.76 mA for 12 weeks and 12 months, respectively.

**Table 3:** Stimulation setting by visit postoperatively.

	Postoperative visit time					
	24 h	10–20 d	6 wk	12 wk	6 mo	12 mo
Number of active leads—n/N (%)						
None	0/15 (0.0%)	0/15 (0.0%)	2/15 (13.3%)	2/15 (13.3%)	5/15 (33.3%)	2/15 (13.3%)
One lead	0/15 (0.0%)	0/15 (0.0%)	0/15 (0.0%)	0/15 (0.0%)	0/15 (0.0%)	0/15 (0.0%)
Two leads	1/15 (6.7%)	1/15 (6.7%)	1/15 (6.7%)	1/15 (6.7%)	1/15 (6.7%)	1/15 (6.7%)
Three leads	1/15 (6.7%)	1/15 (6.7%)	2/15 (13.3%)	2/15 (13.3%)	1/15 (6.7%)	2/15 (13.3%)
Four leads	13/15 (86.7%)	13/15 (86.7%)	10/15 (66.7%)	10/15 (66.7%)	8/15 (53.3%)	10/15 (66.7%)
Frequency (Hz)						
N	57	57	48	48	37	48
Mean ± SD	20.0 ± 0.00	20.0 ± 0.00	20.0 ± 0.00	20.0 ± 0.00	20.0 ± 0.00	20.0 ± 0.00
Pulse width (μs)						
N	57	57	48	48	37	48
Mean ± SD	250.9 ± 6.62	250.9 ± 6.62	250.0 ± 0.00	250.0 ± 0.00	250.0 ± 0.00	250.0 ± 0.00
Amplitude (mA)						
N	57	57	48	48	37	48
Mean ± SD	0.4921 ± 0.4689	0.7081 ± 0.8262	0.5927 ± 0.6192	0.7047 ± 0.8719	0.9081 ± 1.0999	0.7604 ± 1.0546
Median	0.3750	0.4500	0.3750	0.3500	0.4500	0.4250
Min, Max	0.050, 2.400	0.025, 3.925	0.050, 3.125	0.075, 4.450	0.050, 4.175	0.050, 4.800

### Primary safety endpoint

Patients were followed up to 1 year postoperatively and underwent clinical assessment related to the safety of the combined approach including AEs related to the device, surgical procedure, stimulation therapy, and/or TLIF and DRG stimulation combination. A total of 16 related AEs in 9 subjects were reported by the investigational sites (Table 4). None of the 16 related AEs was classified as serious AE by the investigators. The majority of the related AEs were nervous system disorder in 6 subjects (26.7%) and general disorders and administration site conditions in 3 subjects (13.3%). All related AEs were anticipated to this type of surgical procedure and the occurrence of the AEs was within the standard-of-care incidence. None of the related AEs were due to the 2 procedures, TLIF and DRG stimulation, being combined.

**Table 4:** Summary of all related adverse event (AE).

System organ class/Preferred term	Events	Subjects (N = 15)
All device/surgical procedure/stimulation therapy related AEs	16	9/15 (60.0%)
Nervous system disorders	6	4/15 (26.7%)
Hypoaesthesia	3	3/15 (20.0%)
Paraesthesia	3	3/15 (20.0%)
General disorders and administration site conditions	3	2/15 (13.3%)
Implant site pain	2	2/15 (13.3%)
Pyrexia	1	1/15 (6.7%)
Blood and lymphatic system disorders	1	1/15 (6.7%)
Anemia	1	1/15 (6.7%)
Gastrointestinal disorders	1	1/15 (6.7%)
Constipation	1	1/15 (6.7%)
Injury, poisoning and procedural complications	1	1/15 (6.7%)
Wound dehiscence	1	1/15 (6.7%)
Musculoskeletal and connective tissue disorders	1	1/15 (6.7%)
Pain in extremity	1	1/15 (6.7%)
Product issues	1	1/15 (6.7%)
Device dislocation	1	1/15 (6.7%)
Renal and urinary disorders	1	1/15 (6.7%)
Urinary retention	1	1/15 (6.7%)
Vascular disorders	1	1/15 (6.7%)
Hypotension	1	1/15 (6.7%)

Of note, the wound dehiscence was categorized by the care provider at 10–20 days follow-up visit (subject 01–15) and not all staples were removed at once due to slow wound healing. Additional follow-up 3 days later was conducted and the remaining staples were removed without actual wound dehiscence. Therefore, there was no actual wound dehiscence for the subject but was still categorized as an AE due to documentation and use of the term by the care provider

Of note, the wound dehiscence was categorized by the care provider at 10–20 days follow-up visit (subject 01–15) and not all staples were removed at once. Additional follow-up 3 days later was conducted and the remaining staples were removed without actual wound dehiscence or issues. Therefore, there was no actual wound dehiscence for the subject but was still categorized as an AE due to documentation and use of the term by the care provider. More appropriate category would be slow wound healing.

Follow-up also included routine anterior-posterior (AP) and lateral x-rays to evaluate the electrode position at regular intervals through 12-months post-implant. There were no postoperative complications related to the device, including wound infection, wound breakdown, device failure/malfunction or loss of stimulation therapy. No surgical revisions were required in all 15 subjects through 12 months follow-up.

AP/lateral X-rays were performed and reviewed by the investigators for device position at all protocol required follow-up visits. Out of the 58 implanted leads, there was one lead migration at 6 weeks postoperatively reported. Subject 01–15 had an X-ray examination at 6 weeks follow-up. Examination of the x-ray revealed that one of the stimulator leads changed from last X-ray. The investigator classified the event as definitely related to DRG stimulator system. The lead was turned off and no longer used.

### Primary efficacy endpoint and secondary endpoint

Change in back pain and leg pain VAS scores from baseline to 12 weeks, 6 months, and 12 months postoperatively was tracked. The mean changes (improvement) in back pain VAS score (Table 5) and leg pain VAS score (Table 6) from baseline to 12 weeks were 4.68 and 4.66, respectively. The back pain and leg pain reductions started at 10–20 days visit and continued to improve through 6- and 12-weeks and 6- and 12-months. At 12 months post-implant, the mean changes of back pain and leg pain VAS scores were 5.1 and 4.7, respectively, which represent 71.0% and 68.6% improvement, respectively.

**Table 5:** Summary of back pain visual analog scale (VAS) score and improvement postoperatively by visit.

	Baseline	10–20 d	6 wk	12 wk	6 mo	12 mo
Back pain VAS score						
N	15	15	14	14	15	15
Mean ± SD	7.06 ± 1.265	3.72 ± 3.045	2.80 ± 2.389	2.35 ± 2.026	1.90 ± 2.058	1.98 ± 2.046
Median	7.50	3.30	2.69	2.00	1.40	1.40
Min, Max	4.8, 8.9	0.0, 8.7	0.0, 7.7	0.0, 6.0	0.0, 6.4	0.0, 5.8
Improvement of back pain VAS score from baseline						
N		15	14	14	15	15
Mean ± SD		3.34 ± 2.990	4.33 ± 2.622	4.68 ± 2.151	5.16 ± 2.490	5.08 ± 2.366
Median		4.40	4.75	5.05	5.30	5.20
Min, Max		−2.1, 8.1	−1.9, 7.6	−0.2, 7.9	−0.6, 8.7	1.3, 8.2
Percentage improvement of back pain VAS score from baseline (%)						
N		15	14	14	15	15
Mean ± SD		47.34 ± 42.267	59.81 ± 37.861	66.63 ± 29.668	72.00 ± 32.021	71.02 ± 29.380
Median		55.77	65.22	70.33	77.33	82.50
Min, Max		−35.4, 100.0	−32.0, 100.0	−2.9, 100.0	−9.7, 100.0	27.1, 100.0

Improvement from baseline is calculated by subtracting postoperative values from baseline values.

**Table 6:** Summary of leg pain visual analog scale (VAS) score and improvement postoperatively by visit.

	Baseline	10–20 d	6 wk	12 wk	6 mo	12 mo
Leg pain VAS score						
N	15	15	15	14	15	15
Mean ± SD	6.83 ± 1.131	3.23 ± 2.548	2.55 ± 2.758	2.30 ± 2.235	1.95 ± 2.581	2.09 ± 2.210
Median	6.70	2.60	1.30	2.15	0.30	2.00
Min, Max	4.9, 8.6	0.0, 8.8	0.0, 7.8	0.0, 6.2	0.0, 7.1	0.0, 6.7
Improvement of leg pain VAS score from baseline						
N		15	15	14	15	15
Mean ± SD		3.60 ± 2.584	4.28 ± 3.006	4.66 ± 2.404	4.88 ± 3.027	4.74 ± 2.348
Median		4.20	5.40	5.65	6.00	4.70
Min, Max		−1.1, 7.0	−0.8, 8.6	0.8, 8.2	−0.6, 8.3	0.7, 8.3
Percentage improvement of leg pain VAS score from baseline (%)						
N		15	15	14	15	15
Mean ± SD		52.03 ± 38.019	61.38 ± 40.591	66.53 ± 33.044	69.14 ± 42.156	68.58 ± 32.742
Median		65.67	79.25	74.45	95.52	70.15
Min, Max		−14.3, 100.0	−11.8, 100.0	11.2, 100.0	−11.8, 100.0	14.3, 100.0

Improvement from baseline is calculated by subtracting postoperative values from baseline values.

Change in neurological status, disability, quality of life, and use of analgesics from baseline to 12 weeks, 6 months and 12 months postoperatively were tracked. A standard-of-care physical exam was performed at every study visit and the neurological status was based on the neurological component of the standard physical exam. There were 5 incidents of abnormal neurological examination in 4 subjects. The subject 01–06 complained about right fingers still having weird sensation and slight numbness in left posterior arm at the 10–20 days visit. This was related to the patient’s prone positioning for the surgery and not directly related to the surgical procedure or the device. The subject also complained about tingling occurring up her leg which woke her up from sleep. This was due to the stimulation and the DRG stimulator was turned down a few days prior to the 6 months visit appointment. Both of the symptoms resolved. The subject 01–07 also complained about numbness in the left arm distal to the elbow and including the third and fifth digits which was again related to patient’s prone positioning for the surgery and not directly related to the surgical procdure or the device. The symptom resolved without any intervention. The subject 01–17 complained about 7/10 right leg pain that seem to be similar to the “nerve pain and burning pain.” No back pain was reported. This was exacerbation of the preoperative radiculopathy which resolved. At the end of the 12 month follow-up, there were no change in the neurological status from baseline.

The Oswestry Disability Index (ODI), a validated outcome measure used in patients with chronic low back pain, was used to track patients’ disability. The questionnaire was self-administered, and scores were associated with degree of disability ranging from minimal to bedbound. The scoring system included a description of degrees of disability relating to scores on the ODI. Scores from 0% to 20% indicated minimal disability; 20% to 40%, moderate disability; 40% to 60%, severe disability; 60% to 80%, crippled; and 80% to 100%, bedbound or exaggerating.

Table 8 presents the mean ODI score for all subjects at baseline and subsequent follow-up visits through 12 months. The mean baseline ODI was 52.6. The mean improvement (reduction in disability) for subjects from baseline to 12 weeks in the ODI score was 25.1 points, which represents a 42.8% improvement. This improvement continued to increase to 47.6% and 52.8% for 6 months and 12 months post-implant, respectively.

The distribution of ODI categories changed over the 12 months postoperatively (Fig. 4). At baseline, 86.6% of the subjects had severe disability or crippled conditions. This decreased to 53.4% at 10–20 days after surgery. Subsequently, there were no crippled conditions category from 6 weeks to 12 months post-implant compared to 33.3% of the cases at baseline. Minimal disability and moderate disability cases were 6.7% for both at baseline and increased to 46.7% and 40.0% at 12 months post-implant, respectively. Severe disability cases were reduced from 53.3% to 13.3% at 12 months post-implant. Therefore, at 12 months post-implantation, minimal and moderate disability increased to 86.7% of patients from 13.4% of patients at baseline.

The Patient-Reported Outcomes Measurement Information System (PROMIS)-10 Global Health Measures were used to assess an individual’s physical, mental, and social health. A T-score of 50 represents the mean score of the general population, and higher scores indicate better physical and mental health. At baseline, the physical health T-score was 34.9. The physical T-scores were 48.1, 47.3 and 46.6 for 12 weeks, 6 months and 12 months, respectively, which is very close to the mean of the general population of 50. The mental health T-scores improved in a similar pattern through 12 months.

The five-level EuroQol five-dimensional questionnaire (EQ-5D-5L), comprised of 5 dimensions (mobility, self-care, usual activities, pain/discomfort and anxiety/depression) with each dimension having 5 levels (no problems, slight problems, moderate problems, severe problems and extreme problems). The subjects were asked to indicate his/her health state by ticking the box next to the most appropriate statement in each of the 5 dimensions. This decision results in a 1-digit number that expressed the level selected for that dimension. The digits for the 5 dimensions are then combined into a 5-digit number that describes the patient’s health state that is calculated to produce the index score. The maximum index score of 1.0 indicates the best health state. At baseline, the EQ-5D-5L index score was 0.32. Post-implant, the score steadily increased to 0.66, 0.69, 0.71, and 0.70 for 6 weeks, 12 weeks, 6 months and 12 months, respectively. Compared to the baseline, these represent 27.2%, 109.2%, 112.5% and 167.3% improvements, respectively.

The EuroQol (EQ) VAS recorded the patient’s self-rated health on a vertical VAS from 100 to 0, where the endpoints were labelled ‘The best health you can imagine’ (100) and ‘The worst health you can imagine’ (0). At the baseline, the EQ VAS score was 61.6. The score increased to over 75 through 12 months except for 6 months, which was 69.1.

**Fig. 3:**
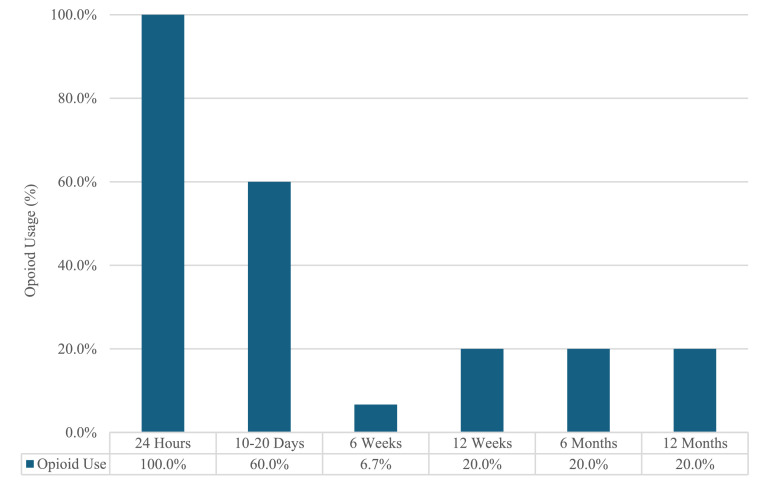
Summary of opioid usage following the procedure.

As expected, 100% of the subjects had opioid medication(s) immediate (24 hours) postoperatively. The opioid usage was down to 20% from 12 weeks to 12 months post-implant (Table 7). Note that subject 01–12 experienced an abdominal pain due to kidney stone at 75 days post-implant and an opioid medication was prescribed. Fig. 3 depicts graphically the trend of opioid use through 12 months.

**Table 7:** Summary of analgesics use from 24 hours postoperatively to 12 months postoperatively.

	24 h	10–20 d	6 wk	12 wk	6 mo	12 mo
Any pain medication	15/15 (100.0%)	12/15 (80.0%)	4/15 (26.7%)	8/15 (53.3%)	7/15 (46.7%)	9/15 (60.0%)
Nonopioid pain medication	10/15 (66.7%)	8/15 (53.3%)	3/15 (20.0%)	6/15 (40.0%)	4/15 (26.7%)	7/15 (46.7%)
Opioid pain medication	15/15 (100.0%)	9/15 (60.0%)	1/15 (6.7%)	3/15 (20.0%)	3/15 (20.0%)	3/15 (20.0%)

**Table 8:** Summary of Oswestry disability index (ODI) by visit postoperatively.

	Baseline	10–20 d	6 wk	12 wk	6 mo	12 mo
ODI score (%)						
N	15	15	14	15	13	15
Mean ± SD	52.6 ± 12.60	45.4 ± 17.18	36.4 ± 15.95	27.5 ± 18.98	27.0 ± 18.48	23.1 ± 16.62
Median	54.0	42.0	38.9	24.4	26.7	30.0
Min, Max	18, 66	26, 78	10, 58	0, 60	0, 60	0, 50
Change from baseline in ODI score (%)						
N		15	14	15	13	15
Mean ± SD		−7.2 ± 21.98	−17.1 ± 22.96	−25.1 ± 22.72	−25.8 ± 20.78	−29.5 ± 20.47
Median		−6.0	−14.8	−24.4	−20.0	−28.0
Min, Max		−38, 36	−52, 31	−66, 8	−66, −4	−64, 0
Percentage change from baseline in ODI score (%)						
n		15	14	15	13	15
Mean ± SD		−2.66 ± 64.470	−20.29 ± 63.358	−42.75 ± 40.393	−47.57 ± 31.644	−52.77 ± 33.417
Median		−15.00	−25.72	−50.00	−44.83	−48.28
Min, Max		−59.7, 200.0	−83.9, 175.0	−100.0, 37.5	−100.0, −6.9	−100.0, 0.0

Change from baseline is calculated by subtracting postbaseline values from baseline values.

**Fig. 4:**
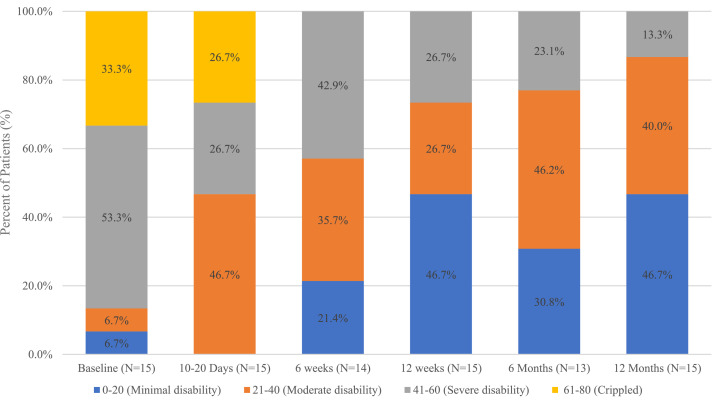
Oswestry Disability Index category changes through the 12 months.

## Discussion

This proof-of-concept pilot series validates our hypothesis that the integrated approach for open DRG stimulator placement at the time of fusion is feasible and advantageous compared to the percutaneous approach to the epidural space, because the DRG electrodes are placed under direct vision of the neural foramen. There is a learning curve in applying a new surgical technique. We made adaptations in the handling of the electrodes without the guide sheath and with respect to the number of leads placed as the patient series progressed. We also observed that with the exposure provided by the spinal approach for laminectomy and decompression, the electrode arrays can be placed on the DRG at the level of the surgery and at the level below the surgery without any difficulty.

DRG lead migration and lead fracture are a common complication for DRG stimulator implantation. For our study, which utilized the Direct Visual Placement of the lead, along with anchoring of the lead to the contralateral rod, there was only 1 incident of lead migration, which was turned off at 6 weeks post-implant, out of 58 total implanted stimulation leads (1.7% per lead and 6.6% per subject). Overall, radiographic imaging after implantation confirmed the relative stability of the DRG leads within the foramen. The one migrated lead was detected on a scheduled surveillance imaging and there were no reports of loss of stimulation benefit.

A study similar in size to our study (57 leads in 19 patients) where the DRG leads were placed percutaneously had a lead migration rate of 15.8% per lead and 31.6% per subject, rate that is much higher than our study [14]. Lead migration using percutaneous implantation techniques for DRG stimulation is a risk, as shown by a multicenter pooled data analysis, with a rate between 2.7% per subject (1.4% per lead) for anchored leads and 21.0% per subject (8.4% per lead) for unanchored leads, even in the hands of experienced implanters [15]. Of note, there was no lead fracture or device failure reported in this study. The lead fracture rates reported by the above multicenter pooled data analysis were 1.9% per lead and 4.8% per subject [15].

Anchoring the electrodes directly to the contralateral fixation rods allowed us to eliminate the use of strain relief loops in the leads that are typically needed when these are introduced percutaneously to the epidural space, and provides for direct, secure fixation of the leads close to their entry points to the foramen. Because the fusion instrumentation is fixed and not mobile with respect to the bone and the neural foramen at the level of the fusion, there is less concern for movement of the leads and therefore minimal concern for lead migration or pull-out. Although anchoring the leads as described should provide additional security and reduced the incidence of lead migration, it can still occur, as the study showed. Therefore, our study demonstrates that implanting DRG leads during the same spinal fusion procedure using direct visual placement of the DRG lead with open approach is feasible and does not negatively affect the leads in terms of lead migration or lead fracture.

Variability between the cases is attributed to surgical learning but also some facility workflow challenges. The first case involved 2 DRG leads being placed while all subsequent cases had 4 leads placed. It was initially thought that leads could only be placed at the index level. However, after completion of the first surgery it was assessed that implant of 2 additional leads at the adjacent caudal level was possible to capture the traversing nerve roots. This would allow the treatment of potential pain that might have been caused by injury to the traversing nerves given the proximity of these nerves to the exiting roots at the index level.

Limitations in OR imaging equipment and/or technician availability in some cases delayed final confirmation of leads before moving onto the tunneling and IPG pocket formation steps. In 1 case, a lead needed to be adjusted after initial anchoring requiring cutting of the anchoring suture and then retying, which also served to validate the robustness of the anchoring technique as the lead could not be easily adjusted (Case 5). In another case, the neuromodulation specialist was proctoring the spine surgeon on how to complete the neuromodulation implant portion of the study (Case 6). The entire workflow of fusion and neuromodulation was completed by the spine surgeon for Case 8, representing the potential for future adoption of the technique by spine surgeons.

The current practice of implanting a permanent DRG stimulator implant is to treat neuropathic pain such as complex regional pain syndrome by the Food and Drug Administration. Other neuromodulation therapy such as SCS is usually utilized after the patient has fully recovered from the fusion procedure and it is clear that the fusion has not adequately addressed their pain. Interestingly, DRG cannot be utilized to treat neuropathic pain after spinal surgery because the epidural placement of the electrodes on the nerve roots are difficult if not impossible due to the postsurgical state and scar formation.

Persistent or recurrent pain after spinal surgery is common, ranging from 20%[16,17] to 40% [5], and can stem from various factors, including the development of adjacent segment disease, hardware failure, pseudoarthrosis (failure of the fusion), or nerve irritation, nerve damage and others. Reoperation is common, and sometimes multiple [18,19]. The proof of concept study was a prospective, multicenter, single-arm, nonrandomized study to evaluate the safety and tolerability of DRG stimulation when the neurostimulator is placed during the same procedure as implantation of the spinal fixation with or without interbody cage systems in patients with chronic back and/or leg pain requiring single- or 2-level spinal fusion.

The study results demonstrated that combining spinal fusion surgery with DRG stimulator implantation during the same procedure is safe. Among 16 device-, surgical procedure, stimulation therapy-, and TLIF and DRG stimulation combination-related AEs, none of them were serious AE classified by site principal investigators. There were no serious neurological deficits caused by fusion procedure and/or DRG stimulation. There were no device revisions or explantations during the study. The reported AE types were anticipated to this type of surgical procedure and the occurrence of the events was within the standard-of-care incidence. None of the related AEs were due to the 2 procedures, TLIF and DRG stimulation, being combined.

Programming parameters were intentionally standardized (20 Hz frequency, 250 μs pulse width) to minimize variability and isolate feasibility and safety outcomes in this initial proof of concept study. While individualized programming is a core component of optimized neuromodulation therapy and may yield superior clinical results, the primary objective of the present investigation was to demonstrate system stability, tolerability, and procedural safety over a 12-month follow-up period. Notably, clinically meaningful improvements in pain and functional outcomes were observed despite the use of conservative, protocol-fixed stimulation parameters. These findings support the feasibility of the integrated fusion-plus-DRG stimulation approach and provide justification for future studies incorporating adaptive and individualized programming strategies, which may further enhance therapeutic outcomes.

The primary efficacy endpoint of the study was the change in back pain and leg pain VAS scores from baseline to follow-up post-implant. The study results showed that both back pain and leg pain VAS scores improved through 12 months follow-up post-implant. The back pain and leg pain VAS scores decreased from 7.1 to 2.0 and 6.8 to 2.1 at 12 months post-implant, respectively. Literature review of spinal fusion surgery with various approaches showed the weighted mean back pain and leg pain VAS scores were 6.83 at baseline decreasing to 2.57 at 12 months and 6.34 at baseline decreasing to 1.69 at months, respectively [[20], [21], [22], [23], [24], [25], [26]]. The percent reduction of the back pain and leg pain VAS scores in this study represented 71.0% and 68.6% improvements, respectively. These results are well in line with the clinical publications which showed that VAS pain reduction post-DRG stimulator implantation were in the range of 52%–75% at 12 months post-implant [[27], [28], [29], [30], [31]].

Responder analyses performed by using minimally clinically important difference (MCID) reduction of the VAS score from baseline showed 86.7% and 93.3% responder rates at 12 months post-implant for both back pain and leg pain met the criterion or either back or leg pain met the criterion, respectively. A more conservative responder criterion was reduction of VAS score at least 50% from the baseline [[31], [32], [33]]. By using this criterion, the responder rates were 66.7% and 73.3% for back pain and leg pains at 12 months post-implant, respectively and were 60.0% and 80.0% for both back pain and leg pain met the criterion or either back or leg pain met the criterion, respectively.

Ardeshiri et al. reported that using 50% reduction of VAS score from baseline, the responder rate to was 60.0% after 12 months restorative neurostimulation in a cohort of 261 chronic lower back pain patients [33]. In a randomized (1:1 ratio) trial (N = 198), Kapural et al. compared 2 neurostimulation configurations for chronic back pain and leg pain using 50% or greater reduction of VAS score from baseline [32]. At 12 months, the responder rate was 78.7% for high frequency configuration (10 kHz) and 51.3% for traditional configuration (50 Hz) for back pain and leg pain. It is noteworthy to point out that all randomized 198 subjects (101: 97) in this study had a trial phase and only the “responder” to the trial phase received a permanent implant (90:81).

In addition, the current study used 20 Hz stimulation frequency. The 12-month responder rate of the current study is higher than the results from the traditional frequency stimulations. It is comparable with the results from the high frequency stimulation considering the cohort were preselected as “responder” during the trial phase.

ODI is a common outcome measure in patients with chronic low back pain used as a secondary endpoint of the study. In this cohort, the mean baseline ODI score was 52.6 points, contributed by 53.3% of the subjects in severe disability and 33.3% of the subjects in crippled conditions. Minimal disability and moderate disability cases were 6.7% of the subjects for both at baseline. The mean improvement (reduction in disability) for subjects from baseline to 12 weeks in the ODI score was 25.1 points, which represents a 42.8% improvement. This improvement continued to increase to 27.0 points (47.6% improvement) and 23.1 points (52.8% improvement) for 6 months and 12 months post-implant, respectively. At 12 months post-implant, significant more subjects (86.7%) were in the minimal disability or moderate disability category compared to the baseline (6.7% for both categories).

Furthermore, responder analysis on ODI using the MCID of 12% showed the responder rates were 69.2% and 80.0% for 6 months and 12 months post-implant, respectively. With a similar baseline mean weighted ODI of 51.2 points, analysis of 10 studies showed the weighted ODI score decreased to 20.7 and 16.4 points for 6 months and 12 months, respectively [[20], [21], [22], [23], [24], [25], [26],^31^,^34^,^35^]. Kallewaard et al. reported at 12 months, 62% of the patients were reclassified either minimal or moderate disability [31]. Kupural et al. reported that at 12 months, 62.9% of high frequency stimulation subjects had minimal or moderate disability compared with 45.7% of traditional 50 Hz stimulation subjects [32]. The current study results are in line with the previous study results.

Other pain and quality of life measures, such as PROMIS-10 and EQ-5D-5L, are all trending in the same direction as the VAS and ODI. This trend supports the observations from the VAS and ODI results and demonstrates the soundness of the study results. Postoperative, especially orthopedic surgery, opioid use are very common. A review of inpatient opioid use and discharge patterns after orthopedic procedure showed that 98% of patients were prescribed opioids following surgery [36]. Among surgical specialties, orthopedic and neurosurgery procedures tend to have the highest rates of opioid use, and interventional spinal procedures fall at the intersection of these specialties [37].

In the current study all subjects were prescribed opioid medications within 24 hours postoperatively. The opioid use was at 60% (n = 9) at 10–20 days postoperatively; then maintained at 20% (n = 3) through 12 weeks to 12 months. It is important to note that 4 of the 15 subjects (26.7%) were opioid free after 24 hours and 6 of the 15 subjects (40%) did not have opioid use from 6 weeks to 12 months. None of the subjects had documented opioid use at more than 2 consecutive visits.

A study using one of the nation’s largest commercial insurance databases to assess predictors of long term opioid following lumber fusion surgery showed that in 8,377 underwent lumbar spinal fusion patients, approximately 50% patients used opioids for a total of 3 months after surgery; 40%, 30%, and 17% used opioids for 6, 12, and 24 months, respectively [38]. Dunn et al. used a more strict definition for chronic opioid use, having a prescription for opioids documented in the medication administration record on each of postoperative days 1–3 and in the clinic medication reconciliation at each postoperative visit at the 1-, 6-, and 12-month time points, and found that out of 958 preoperative opioid users, 498 (52.0%) remained chronic users through 12 months [39]. Among the 367 previously opioid-naive patients, 67 (18.3%) became chronic opioid users [39]. None of the patients in this study met the above criteria.

Psychological comorbidities can influence patient-reported outcomes in both spine surgery and neuromodulation. However, this combined approach differs from traditional stand-alone neuromodulation paradigms in that (1) the spinal fusion surgery addresses a structural pain generator, (2) DRG stimulation is deployed as an adjunct intended to mitigate neuropathic components early, and (3) chronicity and disability duration may be shorter than in typical FBSS cohorts. Accordingly, we do not suggest that psychological factors are irrelevant; rather, we hypothesize that the incremental predictive value of a formal preimplant neuromodulation psychological evaluation—beyond standard perioperative assessment—may be attenuated in this integrated fusion-plus-DRG stimulation treatment. This hypothesis requires prospective validation, and we propose that future trials incorporate standardized psychological measures to determine their independent association with outcomes and device satisfaction.

In conclusion, the study results demonstrate that implanting DRG stimulator during spinal fusion surgery is safe. Taken together all performance information, combination of spinal fusion and DRG stimulator implantation procedure reduced postoperative pain, increased quality of life, and reduced opioids use through 12 months postoperatively. The study outcomes warrant a large size pivotal study to validate proposed combination of spinal fusion and DRG stimulator implantation procedure.

### Limitations

The study had a small sample size limiting generalizability of the results. The first 7 procedures were performed by a spine surgeon and a neuromodulation specialist team, while the remaining procedure was performed by a spine surgeon performing both components of the procedure. Therefore, the series cannot reflect the integrated procedure as if performed by a single surgeon, although the eighth procedure and beyond had similar surgical times.

Over half (56%) of the neuromodulation implant time was spent creating the IPG pocket, tunneling the leads to connect to the IPG, and closing the pocket as is done in the traditional IPG implant approach. Taking advantage of the incision already created for the fusion procedure and avoiding a separate incision and lead tunneling presents an opportunity to reduce the implant time in the future. A single incision IPG implant procedure could provide for a more streamlined workflow that could easily integrate into the current fusion surgical procedure, although reduction in IPG size will be required given the location of the incision.

The study is also an uncontrolled case series for proof of concept study. Therefore, the study lacks the proper internal control which should be kept in mind. This study addresses the feasibility and safety of combining DRG stimulator implantation with surgical decompression and fusion surgery by reporting any AEs related to the device, surgical procedure, stimulation therapy, and/or TLIF and DRG stimulation combination. This study is also inadequate in addressing whether or not the primary efficacy endpoint (back pain and leg pain VAS scores) and secondary endpoint (change in neurological status, disability, quality of life, and use of analgesics) could be due to the effects of the combined procedure and not TLIF alone. Based on the results of this study, future multicenter randomized control study may be appropriate to further explore the therapeutic efficacy of the proposed noval approach.

## Conclusion

By incorporating DRG stimulator implantation into the initial open spinal surgery, we have added the ability to modulate neuropathic pain components in addition to addressing the anatomical concerns with fusion surgery. This pilot series shows feasibility and safety of this technique. We also note that early intervention may have pain outcome benefits, although a larger series over a longer term will be required to assess efficacy. Access to the DRG during the open fusion procedure did not add significant risk. There was no evidence of significant lead migration, loss of efficacy, infection, wound healing complications, or lead fracture in the cohort. This combined technique may become useful for patients who have neuropathic pain components or are at risk of developing them. With further exploration of the technique, we will elucidate patient selection for this combined treatment option.

## Authors contribution

**Michael C. Park:** Conceptualization; Data curation; Formal analysis; Funding acquisition; Investigation; Methodology; Project administration; Resources; Supervision; Validation; Visualization; Roles/Writing—original draft; and Writing—review and editing. **Rohan R. Lall:** Conceptualization; Formal analysis; Investigation; Methodology; Validation; and Writing—review and editing. **Deepak Reddy:** Conceptualization; Formal analysis; Investigation; Methodology; Validation; and Writing—review and editing. **Jonathan N. Sembrano:** Conceptualization; Formal analysis; Investigation; Methodology; Validation; and Writing—review and editing. **Steven M. Falowski:** Formal analysis; Supervision; and Writing—review and editing. **Nameer R. Haider:** Formal analysis; Supervision; and Writing—review and editing.

## Funding

The work presented is part of the SynerFuse Inc., industry sponsored proof of concept study which is being performed under an approved Investigational Device Exemption (IDE) G190045 from the Food and Drug Administration (FDA).

## Ethical approval

The device used in the study is an FDA approved device for a different indication than what is being studied.

## Declaration of competing interests

The authors declare the following financial interests/personal relationships which may be considered as potential competing interests: Michael C. Park, M.D., Ph.D. reports financial support was provided by SynerFuse, Inc. Michael C. Park, M.D., Ph.D. reports a relationship with SetPoint Medical Corporation that includes: funding grants. Michael C. Park, M.D., Ph.D. reports a relationship with ShiraTronics, Inc. that includes: funding grants. Michael C. Park, M.D., Ph.D. reports a relationship with NeuroOne, Inc. that includes: consulting or advisory. Michael C. Park, M.D., Ph.D. reports a relationship with Boston Scientific Corporation that includes: speaking and lecture fees. Michael C. Park, M.D., Ph.D. reports a relationship with SynerFuse, Inc. that includes: board membership, equity or stocks, and travel reimbursement. Rohan R. Lall, M.D. reports a relationship with SynerFuse, Inc. that includes: board membership and equity or stocks. Jonathan N. Sembrano, M.D. reports a relationship with SI-BONE Inc that includes: funding grants and travel reimbursement. Jonathan N. Sembrano, M.D. reports a relationship with Medtronic, Inc. that includes: funding grants. Steven M. Falowski, M.D. reports a relationship with Abbott that includes: speaking and lecture fees. Steven M. Falowski, M.D. reports a relationship with Saluda that includes: speaking and lecture fees. Steven M. Falowski, M.D. reports a relationship with ShiraTronics, Inc. that includes: speaking and lecture fees. Steven M. Falowski, M.D. reports a relationship with Aurora that includes: equity or stocks. Steven M. Falowski, M.D. reports a relationship with SPR that includes: equity or stocks. Steven M. Falowski, M.D. reports a relationship with American Society of Pain & Neuroscience that includes: board membership. Michael C. Park, M.D., Ph.D. has patent issued to SynerFuse, Inc. Steven M. Falowski, M.D. has patent DRG Neuromonitoring issued to Steven M. Falowski, M.D. If there are other authors, they declare that they have no known competing financial interests or personal relationships that could have appeared to influence the work reported in this paper.
